# Antibody glycosylation as an immunological key in health and disease

**DOI:** 10.1093/glycob/cwaa017

**Published:** 2020-02-24

**Authors:** Mattias Collin

**Affiliations:** Division of Infection Medicine, Department of Clinical Sciences, Lund University, Biomedical Center B14, SE-22184 Lund, Sweden

Antibodies, or immunoglobulins, are key molecules in the adaptive immune response that serve as both cell-associated and soluble receptors for foreign material. Through these activities immunoglobulins orchestrate effector functions including neutralization of cytotoxic substances, cell mediated responses, development of immunological memory, activation of the complement system and tissue repair and remodeling. The protein backbone of immunoglobulins has been extensively investigated, both the constant domains (Fc) and the hypervariable antigen-binding regions (Fab). This has allowed for the development of monoclonal antibodies (mAb) and also recombinantly expressed antibodies with very defined properties that are used for therapeutic and research purposes. More recently, immunoglobulins have been recognized as glycoproteins, with N-linked and O-linked glycans attached to both the Fab and Fc portions of the molecules. It has become evident that these glycans are not merely decoration, but are functionally important structural components of the immunoglobulins. This knowledge is the result of hard and systematic work by dedicated researchers over many years. However, in recent years, broad interest in immunoglobulin glycosylation has surged, partly as a consequence of the rapidly expanding arsenal of therapeutic antibodies. An example that makes this quite evident is that one of the most cited articles in recent years in *Glycobiology* is an article that discusses the importance of glycosylation in relation to functions of therapeutic mAbs (Reusch and Tejada, 2015). Therefore, it was with great pleasure I accepted the honor to guest edit this special issue on Immunoglobulin Glycosylation, where I was able to invite researchers that have greatly contributed to the increased knowledge in this field and that have inspired me and many others to venture into this field.

The first article is an excellent introduction to the field by Cobb (2019) who gives an in-depth historical account of research on functions of IgG glycosylation from the mid-1970s to today. This review highlights the functional importance of the glycans on IgG and the link between IgG glycosylation and different diseases.

The second review by Yamaguchi and Barb (2019) introduces how glycosylation of the IgG Fc region affects its structure and discusses what is known about the recognition and binding of the *N*-glycan by Fc receptors. The review also discusses some of the technological challenges involved in elucidating the role of Fc glycans in Fc quaternary structure, and highlights technical achievements that have aided in a deepened understanding of Fc structure and motion.

In the third review, de Haan and Wuhrer (2019) describe technical developments that have made possible the recent leap in knowledge of roles for immunoglobulin glycosylation. They cover state-of-the-art techniques for immunoglobulin glycan analysis in complex clinical samples that have allowed investigations into the contributions of immunoglobulin glycosylation to health and disease. They also describe robust and reproducible techniques that can be more routinely used for the analysis of IgG Fc glycosylation in clinical laboratories and during the development of antibody-based drugs.

In the fourth review by Irvine and Alter (2020), immunoglobulin glycosylation is viewed from an infectious disease perspective. They present an overview of recent findings that have revealed immunoglobulin glycosylation dynamics during infections that are keys to understanding pathogenicity, and also give rise to new principles for preventing and treating infectious disease (Irvine and Alter, 2020).

The fifth review by Sjögren et al. (2019) introduces a family of enzymes of bacterial origin with activity on IgG glycans. They exemplify how these can be used for glycan analysis for research or clinical purposes, remodeling of Fc glycans for desired effector functions, increasing specificity in antibody-based imaging and generating uniform conjugation of molecules to IgG Fc for imaging or as antibody-drug conjugates.

In the sixth and final review, Du et al. (2019) showcase the structural biology behind the glycan and protein specificity of IgG-active endoglycosidases. They put this is the context of current proposed activities including chemoenzymatic tailoring of Fc glycans on intact antibodies and potential identification and development of additional enzymes with desired activities on Fc glycans.

It is my hope that, taken together, the reviews in this special issue will serve as a “go to” reference for both experienced researchers and newcomers to the field of immunoglobulin glycosylation. Furthermore, I also hope the reviews will inspire immunologists, autoimmunologists, antibody-drug and antigen-dependent cytotoxicity developers, microbiologists and other clinical or basic researchers to start thinking about the functions of immunoglobulin glycosylation. This glycosylation might be a key to understanding pathogenicity, be used as a diagnostic, be exploited to direct effector functions and might be used to deliver other drugs or in other ways contribute to understanding the fundamental biology of the immune system.
